# High-yield production of l-serine through a novel identified exporter combined with synthetic pathway in *Corynebacterium glutamicum*

**DOI:** 10.1186/s12934-020-01374-5

**Published:** 2020-05-29

**Authors:** Xiaomei Zhang, Yujie Gao, Ziwei Chen, Guoqiang Xu, Xiaojuan Zhang, Hui Li, Jinsong Shi, Mattheos A. G. Koffas, Zhenghong Xu

**Affiliations:** 1 Laboratory of Pharmaceutical Engineering, School of Pharmaceutics Science, Jiangnan University, Wuxi, People’s Republic of China; 2 National Engineering Laboratory for Cereal Fermentation Technology, Jiangnan University, Wuxi, People’s Republic of China; 3 The Key Laboratory of Industrial Biotechnology, Ministry of Education, School of Biotechnology, Jiangnan University, Wuxi, People’s Republic of China; 4 Department of Chemical and Biological Engineering, Rensselaer Polytechnic Institute, Troy, NY USA; 5 Department of Biological Sciences, Rensselaer Polytechnic Institute, Troy, NY USA

**Keywords:** l-Serine, Exporter, *C. glutamicum*, Transcriptional regulator, Metabolic engineering

## Abstract

**Background:**

l-Serine has wide and increasing applications in industries with fast-growing market demand. Although strategies for achieving and improving l-serine production in *Corynebacterium glutamicum* (*C. glutamicum*) have focused on inhibiting its degradation and enhancing its biosynthetic pathway, l-serine yield has remained relatively low. Exporters play an essential role in the fermentative production of amino acids. To achieve higher l-serine yield, l-serine export from the cell should be improved. In *C. glutamicum*, ThrE, which can export l-threonine and l-serine, is the only identified l-serine exporter so far.

**Results:**

In this study, a novel l-serine exporter NCgl0580 was identified and characterized in *C. glutamicum* ΔSSAAI (SSAAI), and named as SerE (encoded by *serE*). Deletion of *serE* in SSAAI led to a 56.5% decrease in l-serine titer, whereas overexpression of *serE* compensated for the lack of *serE* with respect to l-serine titer. A fusion protein with SerE and enhanced green fluorescent protein (EGFP) was constructed to confirm that SerE localized at the plasma membrane. The function of SerE was studied by peptide feeding approaches, and the results showed that SerE is a novel exporter for l-serine and l-threonine in *C. glutamicum*. Subsequently, the interaction of a known l-serine exporter ThrE and SerE was studied, and the results suggested that SerE is more important than ThrE in l-serine export in SSAAI. In addition, probe plasmid and electrophoretic mobility shift assays (EMSA) revealed NCgl0581 as the transcriptional regulator of SerE. Comparative transcriptomics between SSAAI and the NCgl0581 deletion strain showed that NCgl0581 is a positive regulator of NCgl0580. Finally, by overexpressing the novel exporter SerE, combined with l-serine synthetic pathway key enzyme *serA*Δ197, *serC,* and *serB*, the resulting strain presented an l-serine titer of 43.9 g/L with a yield of 0.44 g/g sucrose, which is the highest l-serine titer and yield reported so far in *C. glutamicum*.

**Conclusions:**

This study provides a novel target for l-serine and l-threonine export engineering as well as a new global transcriptional regulator NCgl0581 in *C. glutamicum*.

## Background

l-Serine has been identified as one of the top 30 most interesting building blocks for a range of chemicals and materials, and is used in cosmetic, pharmaceutical, and food industries [1, 2]. Metabolic engineering of *C. glutamicum* for l-serine production has been focused on its terminal synthesis pathways and degradation pathways, and proven to be very useful for improving l-serine production in this bacterium [3–6]; however, the l-serine productivity achieved is still low for large-scale l-serine production, and the highest reported l-serine titer is 42.62 and 50 g/L with an yield of 0.21 g/g sucrose and 0.36 g/g glucose in *C. glutamicum* and *Escherichia coli* (*E. coli*), respectively. Besides, l-serine can also be potentially produced from sugar via fermentation with a very high theoretical yield (1.17 g/g glucose, 1.22 g/g sucrose) [2].

For enhancing l-serine production, an improvement in l-serine export from the cell should be considered. Export system plays an essential role in metabolic engineering strategies for the production of amino acids [7], because it reduces intracellular amino acid concentrations, thereby alleviating feedback inhibition and circumventing toxicity problems [1, 8–10]. In recent decades, several export systems have been identified for excreting amino acids, such as l-lysine, l-cysteine, L-glutamate, l-threonine, l-arginine, l-methionine, and branched-chain amino acids, in *C. glutamicum* and *E. coli* [11–17]. However, to the best of our knowledge, except for ThrE (l-threonine and l-serine exporter) [15, 18], no other l-serine exporters have been reported in *C. glutamicum* so far. In *E. coli*, Mundhada et al. found that intracellular l-serine accumulation was toxic to the engineered strain modified to produce l-serine, and that following overexpression of *eamA*, which encodes l-cysteine exporter in *E. coli*, the engineered strain exhibited increased tolerance toward l-serine with higher l-serine productivity [2]. Therefore, l-serine exporter in *C. glutamicum* could be a potential target for strain optimization to further improve l-serine production.

It has been reported that homologs similar to the exporters in *E. coli* might fulfil a comparable function in *C. glutamicum* [17, 19, 20]. Accordingly, we hypothesized that the homolog to EamA(l-serine exporter in *E. coli*) might be involved in l-serine export in *C. glutamicum*. In the present study, three homologs to EamA, namely, NCgl2050, NCgl2065, and NCgl0580, were determined, and their functions were identified by targeted gene deletion, respectively. The results showed that one of the genes, NCgl0580 gene, was involved in l-serine export. Subsequently, localization and function of NCgl0580 were investigated, and the interaction of a known l-serine exporter ThrE (encoded by *thrE*) and the novel exporter NCgl0580 was studied. Furthermore, the transcriptional regulator of NCgl0580 was identified and studied. Finally, the effects of overexpression of l-serine exporter in combination with l-serine synthetic pathway enzyme on l-serine production were evaluated.

## Results

### Exploring putative l-serine exporters in *C. glutamicum*

In previous studies, homologs of *E. coli* exporters have been shown to have similar functions in *C. glutamicum* [17, 19, 20]. Therefore, we hypothesized that the *C. glutamicum* homolog to EamA(l-serine exporter in *E. coli*) [2] might be involved in l-serine export in *C. glutamicum*. According to the NCBI database, EamA belongs to the RhaT superfamily, and 15 records of related proteins associated with RhaT superfamily in *C. glutamicum* ATCC13032 were obtained. After eliminating duplicate records, three related genes, NCgl2050, NCgl2065, and NCgl0580 genes, were obtained, which might be involved in l-serine export in *C. glutamicum*.

**Fig. 1:**
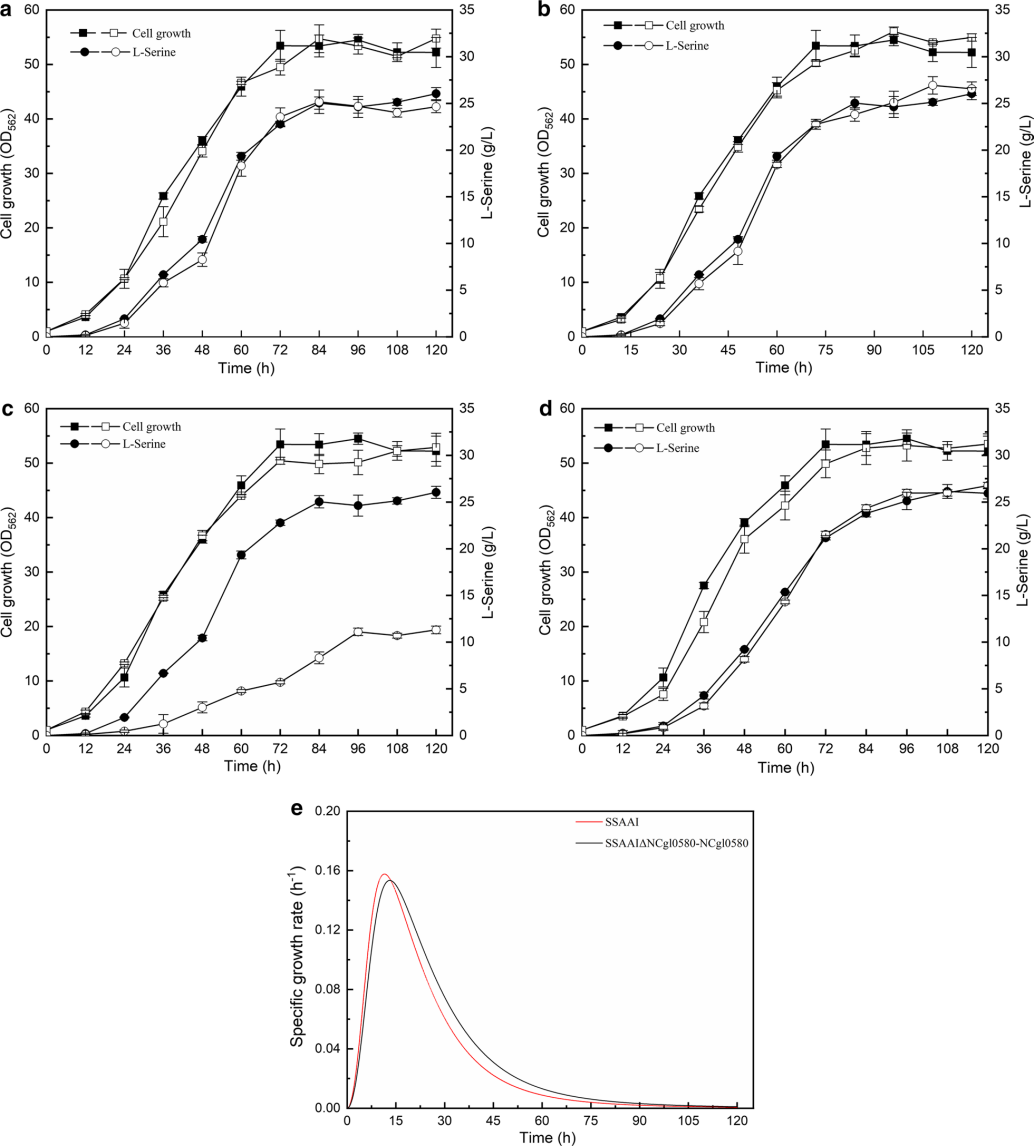
Effect of NCgl2050, NCgl2065, and NCgl0580 deletion and NCgl0580 complement on SSAAI. **(a)** Cell growth (squares) and l-serine titer (circles) of NCgl2050 deletion strain SSAAIΔNCgl2050 (open symbols) and SSAAI (solid symbols). **b** Cell growth (squares) and l-serine titer (circles) of NCgl2065 deletion strain SSAAIΔNCgl2065 (open symbols) and SSAAI (solid symbols). **c** Cell growth (squares) and l-serine titer (circles) of NCgl0580 deletion strain SSAAIΔNCgl0580 (open symbols) and SSAAI (solid symbols). **d** Cell growth (squares) and l-serine titer (circles) of complemented strain SSAAI∆NCgl0580-NCgl0580 (open symbols) and SSAAI (solid symbols). Squares and circles indicate cell growth OD_562_ and l-serine titer, respectively. **e** The growth rates of the complemented strain SSAAI∆N0580-NCgl0580 (red) and SSAAI (black)

To verify the function of these putative proteins in *C. glutamicum* SSAAI (SSAAI), NCgl2050, NCgl2065, and NCgl0580 were deleted in this strain respectively. The results showed that the deletion of NCgl2050 and NCgl2065 did not produce any changes in cell growth and l-serine titer (Fig. 1a, b). Strikingly, deletion of NCgl0580 significantly reduced the l-serine titer in SSAAI, but did not affect the growth of the strain (Fig. 1c). SSAAI ΔNCgl0580 produced 11.31 g/L l-serine, which was 56.5% (p < 0.001) lower than that noted in SSAAI (Fig. 1c). However, plasmid-borne overexpression of NCgl0580 compensated for the lack of NCgl0580 with respect to l-serine titer, resulting in 26.76 g/L l-serine titer, similar to that generated by the parent strain SSAAI (Fig. 1d). As shown in Fig. 1d, e, when compared with SSAAI, the strain harboring the plasmid grew slowly to some extent in the logarithmic growth phase, finally reaching cell growth similar to that of SSAAI. This finding suggested that NCgl0580 might act as the l-serine exporter in *C. glutamicum*, and was named as SerE and its function was further investigated.

### Localization and function of SerE

According to the NCBI, SerE was presumed to be a hypothetical membrane protein of 301 amino acids, similar to permease of the drug/metabolite transporter (DMT) superfamily. The transmembrane helices of SerE were predicted by TMHMM Server v. 2.0, and SerE exhibited ten transmembrane-spanning helices with both amino- and carboxy-terminal ends in the cytoplasm.

**Fig. 2:**
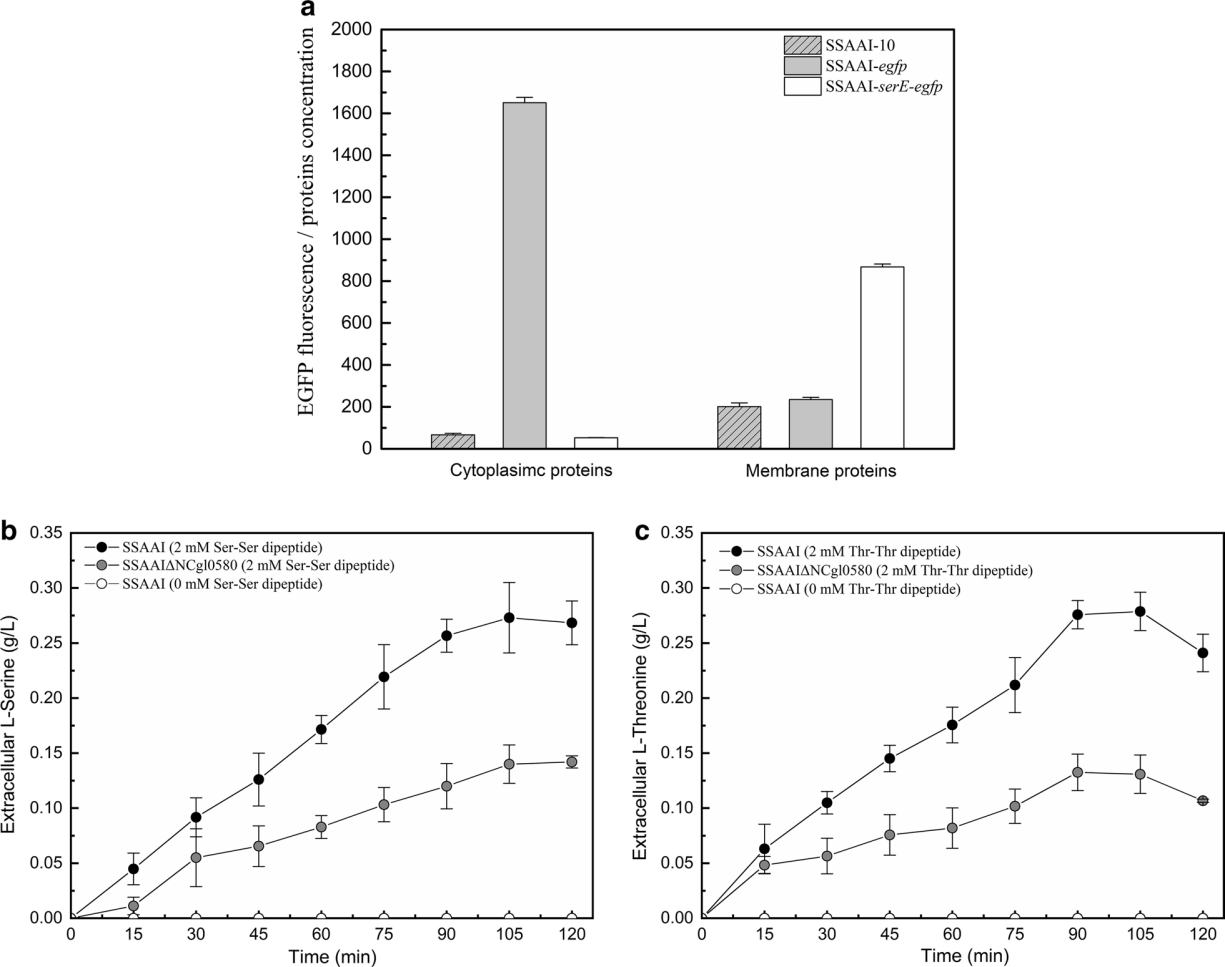
Fluorescence of cytoplasmic proteins and membrane proteins, and the result of amino acid export of SerE by using peptide feeding approach in SSAAI. **a** Fluorescence of cytoplasmic proteins and membrane proteins of SSAAI-10 (SSAAI harboring plasmid pDXW-10 only, gray bar with slash), SSAAI-*egfp* (SSAAI overexpressing EGFP protein with pDXW-10, gray bar), and SSAAI-*serE*-*egfp* (SSAAI overexpressing SerE-EGFP fusion protein with pDXW-10, white bar). **b** Extracellular concentration of l-serine in SSAAI (black circles) and *serE* deletion strain SSAAI Δ*serE* (gray circles) with 2 mM of the dipeptide Ser–Ser. Extracellular concentration of l-serine in SSAAI (white circles) without the dipeptide Ser–Ser. **c** Extracellular concentration of l-threonine in SSAAI (black circles) and serE deletion strain SSAAI Δ*serE* (gray circles) with 2 mM of the dipeptide Thr–Thr. Extracellular concentration of l-threonine in SSAAI (white circles) without the dipeptide Thr–Thr

To confirm the localization of SerE, SerE-EGFP fusion protein was expressed in SSAAI. Confocal microscopic observations of SSAAI-*egfp* and SSAAI-*serE*-*egfp* confirmed that EGFP and SerE-EGFP fusion proteins were successfully expressed, respectively (Additional file 1: Fig. S1). To further verify the localization of SerE, membrane and cytoplasmic proteins from these two strains were extracted by ultrasonication, and the fluorescence of these proteins was determined using a fluorescence spectrophotometer. The fluorescence of the cytoplasmic proteins of SSAAI-*egfp* and membrane proteins of SSAAI-*serE*-*egfp* (Fig. 2a) affirmed that SerE was localized at the plasma membrane in SSAAI.

To substantiate the function of SerE, a peptide feeding approach was employed by incubating SSAAI and SerE deletion strain, SSAAI Δ*serE,* with 2 mM of the dipeptide Ser–Ser, respectively, and measuring the concentration of extracellular l-serine. As shown in Fig. 2b, a higher l-serine concentration was detected in SSAAI, when compared with that in SSAAI Δ*serE*, confirming that SerE is a novel exporter of l-serine in *C. glutamicum*.

It is known that l-cysteine export system in *E. coli* (encoded by *eamA*) also catalyzes l-serine export [2], and that l-threonine exporter in *C. glutamicum* (encoded by *thrE*) also transports l-serine [15]. We therefore analyzed whether the novel exporter SerE could export l-cysteine or l-threonine. The export experiments with dipeptides (Thr–Thr, Cys–Cys) were performed using SSAAI and SSAAI Δ*serE*. The dipeptides were added at a concentration of 2 mM to the medium, and the extracellular amino acid concentrations at different time intervals were determined by HPLC. The results revealed that the concentration of l-cysteine was comparable in both strains and did not significantly change (data not shown), indicating that SerE might not export l-cysteine. Interestingly, the concentrations of l-threonine in SSAAI Δ*serE* were lower than those in SSAAI (Fig. 2c), indicating that SerE might be also an exporter of l-threonine in *C. glutamicum*.

### Interaction of a known exporter ThrE and a novel exporter SerE

**Fig. 3:**
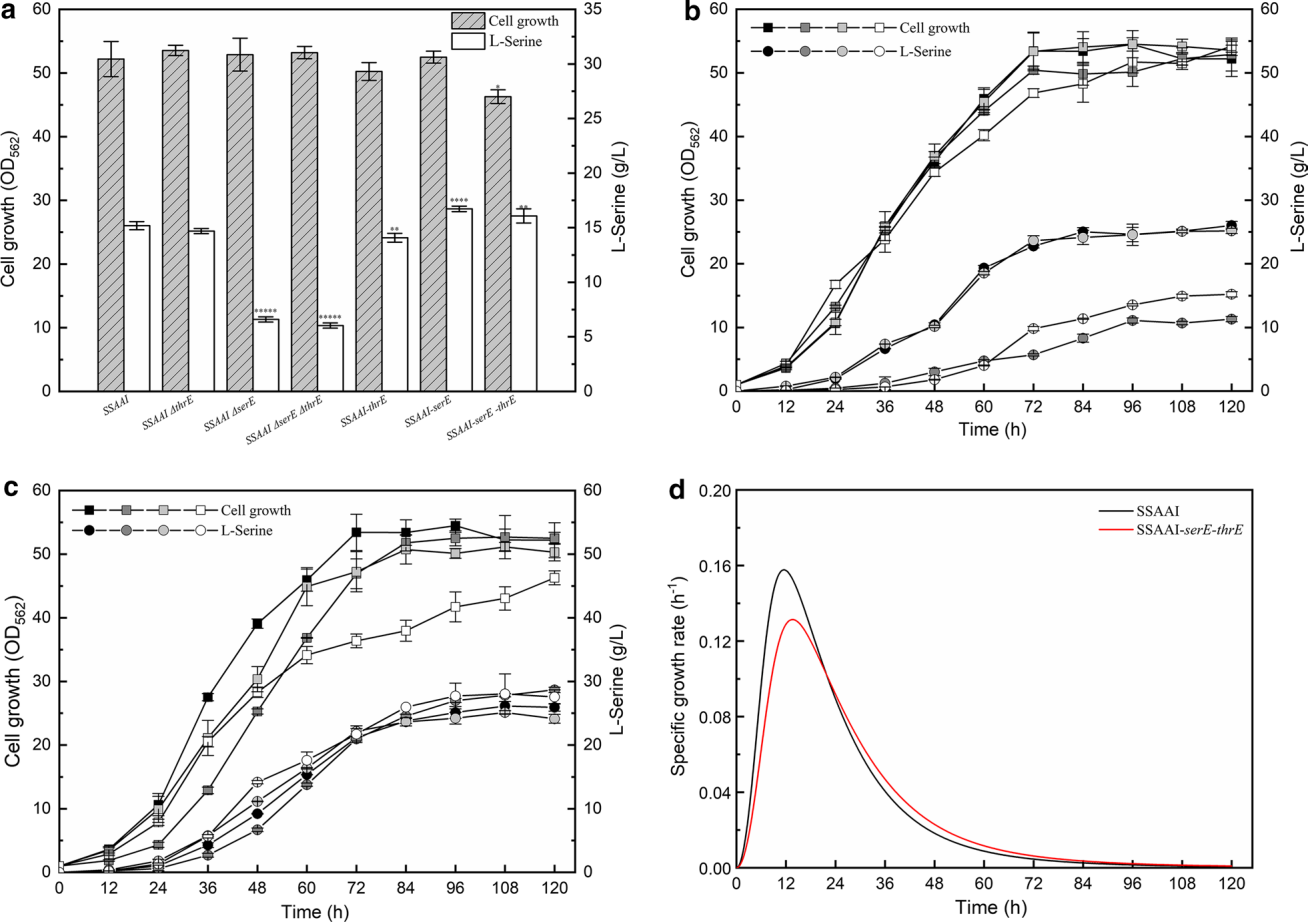
Effect of the exporters *thrE* and *serE* deletion or overexpression on SSAAI. **a** Cell growth (gray bar with slash) and l-serine titer (white bar) of SSAAI, *thrE* deletion strain SSAAI ∆*thrE*, serE deletion strain SSAAI ∆*serE*, *thrE* and *serE* deletion strain SSAAI ∆*serE* ∆*thrE*, *thrE* overexpression strain SSAAI-*thrE*, *serE* overexpression strain SSAAI-*serE*, and *thrE* and *serE* double overexpression strain SSAAI-*serE* -*thrE*. **b** Cell growth (squares) and l-serine titer (circles) of SSAAI (black), *serE* deletion strain SSAAI ∆*serE* (dark gray), *thrE* deletion strain SSAAI ∆*thrE* (gray), and *thrE* and *serE* deletion strain SSAAI ∆*serE* ∆*thrE* (white). **c** Cell growth (squares) and l-serine titer (circles) of SSAAI (black), *serE* overexpression strain SSAAI-*serE* (dark gray), *thrE* overexpression strain SSAAI-*thrE* (gray), and *thrE* and *serE* double overexpression strain SSAAI-*serE*-*thrE* (white). **d** The growth rates of SSAAI-*serE*-*thrE* (red) and SSAAI (black)

It is well known that *thrE* encodes ThrE that can export l-threonine and l-serine in *C. glutamicum* ATCC13032 [15]. To understand the interaction between ThrE and SerE on l-serine export, *thrE* was deleted in SSAAI (SSAAI Δ*thrE),* which did not produce any significant change in l-serine titer in the deletion mutant (Fig. 3a, b). In contrast, deletion of SerE significantly reduced the l-serine titer in SSAAI, and resulted in little change in cell growth (Fig. 1c). The SSAAI Δ*serE* produced 11.31 g/L l-serine, which was 56.5% (p < 0.001) lower than that produced by SSAAI (Fig. 1c). Subsequently, *thrE* and *serE* double deletion mutant was constructed, which exhibited cell growth comparable to that of SSAAI, and produced 10.34 g/L l-serine, which was 60% (p < 0.001) lower than that observed in SSAAI (Fig. 3a, b).

Furthermore, *thrE* and *serE* were overexpressed alone or in combination in SSAAI to obtain SSAAI-*thrE,* SSAAI-*serE,* and SSAAI-*serE*-*thrE*. While l-serine accumulation in SSAAI-*thrE* was similar to that in SSAAI, the production of l-serine in SSAAI-*serE* reached 28.67 g/L, which was 10.5% (p < 0.01) higher than that noted in SSAAI (Fig. 3a, c). However, a decrease in cell growth was observed in SSAAI-*serE* before 72 h of fermentation, when compared with that found in SSAAI (Fig. 3d). Furthermore, no significant difference in l-serine titer was found in the time courses of both SSAAI-*serE* and SSAAI-*serE*-*thrE*, and SSAAI-*serE*-*thrE* exhibited lower cell growth than SSAAI-*thrE* before 96 h of fermentation (Fig. 3c, d). These observations might be due to the inhibition of cell growth resulting from l-serine over-efflux, metabolic burden of overexpression of two membrane-binding proteins, or inhibition of cell growth by l-threonine over-efflux. Taken together, these findings suggested that SerE plays a more important role than ThrE for l-serine export in SSAAI.

### Transcriptional regulator of the novel exporter SerE

The gene NEWCgl0581, located upstream of *serE* and divergently transcribed from *serE* (Additional file 1: Fig. S2), and its product (consisting of 303 amino acids) was found to be a member of the LysR-type transcriptional regulators (LTTRs) family. It must be noted that LTTRs were initially described as regulators of divergently transcribed genes [21]. In a previous study on *C. glutamicum,* LysG, located upstream of l-lysine exporter gene *lysE,* was observed to encode a LysR-type transcriptional regulator, confirming that LysG is a positive transcriptional regulator of *lysE* [22]. Accordingly, we speculated that NCgl0581 might be involved in the control of *serE* transcription.

**Fig. 4:**
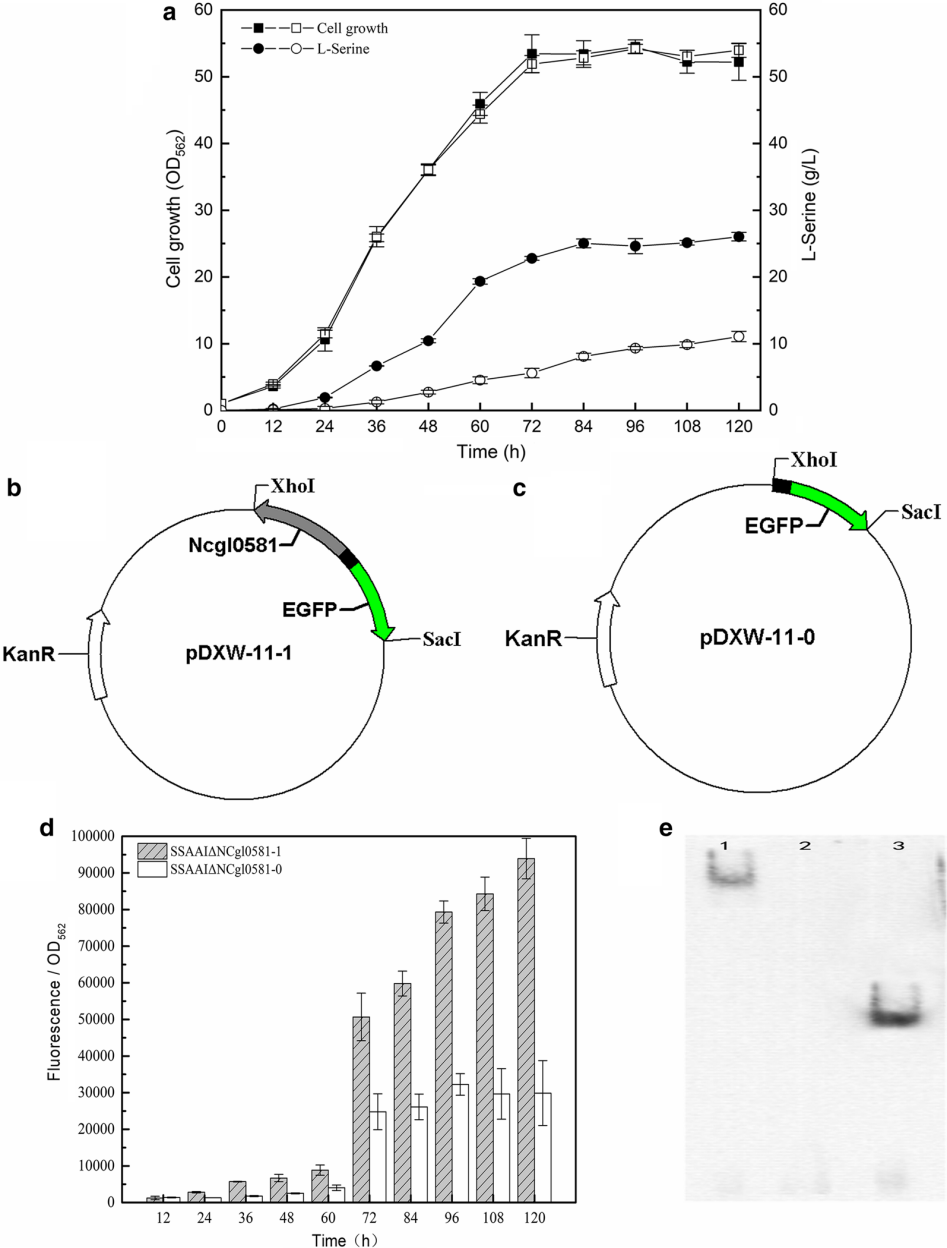
Verification of the function of NCgl0581. **a** The cell growth (squares) and l-serine titer (circles) of SSAAI (solid symbols) and NCgl0581 deletion strain SSAAIΔNCgl0581 (open symbols), respectively. **b** Plasmid pDXW-11-1 containing fragments of NCgl0581 (gray), intergenic region between NCgl0581 and NCgl0580 (black), and EGFP (green). **c** Plasmid pDXW-11-0 containing fragments of the intergenic region between NCgl0581 and NCgl0580 (black) and EGFP (green). **d** Fluorescence of the two strains, SSAAI ΔNCgl0581-1 (gray bar with slash) and SSAAIΔNCgl0581-0 (white bar). **e** Verification of NCgl0581 binding to the upstream region of SerE by using EMSA. Lane 1: the nuclear extracts with activated specific TF (positive control), Lane 2: the nuclear extracts without activated TF (negative control), Lane 3: Sample

To determine the function of NCgl0581, a mutant strain with NCgl0581 deletion was constructed. As shown in Fig. 4a, the growth of SSAAI ΔNCgl0581 was similar to that of the parent strain SSAAI. However, the l-serine titer of SSAAI ΔNCgl0581 was 11.08 g/L, which was 57.4% (p < 0.001) lower than that of the parent strain, indicating that NCgl0581 played an important role in l-serine production. Subsequently, the effect of NCgl0581 on *serE* expression was further investigated by using the probe plasmid pDXW-11. Two recombinant strains, SSAAI ΔNCgl0581-1 (harboring the plasmid pDXW-11-1, Fig. 4b) and SSAAI ΔNCgl0581-0 (harboring the plasmid pDXW-11-0, Fig. 4c) were constructed, and their fluorescence during fermentation was measured. The fluorescence of SSAAI ΔNCgl0581-1 was stronger than that of SSAAI ΔNCgl0581-0 during the fermentation process (Fig. 4d), revealing that NCgl0581 functioned as a positive regulator of *serE* expression. To verify whether the regulatory protein NCgl0581 binds to the upstream region of SerE, EMSA was performed by using the DNA probe labeled with biotin, and the result clearly indicated that NCgl0581 binds to this region (Fig. 4e).

To confirm whether NCgl0581 is a specific regulator of SerE, transcriptome sequencing was performed using SSAAI and NCgl0581 deletion strain. The findings showed that the transcription levels of 115 genes were altered, including 56 upregulated genes and 59 downregulated genes, in response to NCgl0581 deletion, indicating that NCgl0581 is a global transcriptional regulator in *C. glutamicum.* The genes with significant transcriptional change (≥ fourfold) are shown in Tables 1 and 2.

**Table 1:** Genes significantly upregulated by NCgl0581 deletion

Gene id	SSAAI Δ0581	SSAAI	Fold change	Protein function
NCgl2897	701.56	71.07	9.87	Starvation-inducible DNA-binding protein
NCgl0546	17.78	2.75	6.45	Hypothetical protein
NCgl1405	15.94	2.71	5.88	ABC transporter periplasmic component
NCgl1302	10.05	1.96	5.13	Aldo/keto reductase
NCgl1344	286.87	55.96	5.12	Ornithine carbamoyltransferase
NCgl1343	280.65	57.24	4.9	Acetylornithine aminotransferase
NCgl0746	43.30	9.04	4.7	Hypothetical protein
NCgl1342	134.70	29.07	4.63	Acetylglutamate kinase
NCgl2946	672.93	155.87	4.31	Hypothetical protein
NCgl1022	89.53	21.28	4.20	Cysteine sulfinate desulfinase
NCgl1023	368.88	88.67	4.15	Nicotinate-nucleotide pyrophosphorylase
NCgl1341	108.49	27.09	4.00	Bifunctional ornithine acetyltransferase/*N*-acetylglutamate synthase

**Table 2:** Genes significantly downregulated by NCgl0581 deletion

Gene id	SSAAI Δ0581	SSAAI	Fold change	Protein function
NCgl0580	18.40	5152.54	280.02	Hypothetical protein
NCgl0638	1.71	20.97	12.22	ABC transporter permease
NCgl0639	11.00	82.47	7.49	ABC transporter periplasmic component
NCgl2943	207.03	1355.55	6.54	Hypothetical protein
NCgl0943	16.19	103.52	6.39	AraC family transcriptional regulator
NCgl0484	2.32	14.57	6.28	ABC transporter permease
NCgl2942	283.52	1776.15	6.26	NADH:flavin oxidoreductase
NCgl0166	13.41	79.70	5.94	Hypothetical protein
NCgl0324	2.11	11.87	5.61	Zn-dependent alcohol dehydrogenase
NCgl0282	5.19	28.25	5.44	4-Hydroxyphenyl-beta-ketoacyl-CoA hydrolase
NCgl1975	102.94	503.75	4.89	Hypothetical protein
NCgl2893	1.25	6.08	4.84	Efflux system protein
NCgl0155	9.11	43.69	4.79	5-Dehydro-2-deoxygluconokinase
NCgl0014	10.02	47.76	4.76	Hypothetical protein
NCgl2953	7.68	35.80	4.66	Sugar permease
NCgl2744	12.26	55.19	4.50	Hypothetical protein
NCgl2970	15.22	67.51	4.43	ABC transporter periplasmic component
NCgl0608	23.06	100.35	4.35	ABC transporter permease
NCgl0258	4.51	19.50	4.32	Arsenite efflux pump ACR3
NCgl0281	16.83	67.69	4.02	Dehydrogenase

The transcriptional level of *serE* was significantly decreased by 280-fold following NCgl0581 deletion, revealing that NCgl0581 is a positive regulator of *serE*. Furthermore, NCgl0581 deletion downregulated the two ABC transporter permeases (NCgl0638 and NCgl0484) and ABC transporter periplasmic component (NCgl0639) by 12-, 6.3-, and 7.5-fold, respectively, and upregulated ABC transporter periplasmic component (NCgl1405) by 5.88-fold, suggesting that NCgl0581 is involved in the synthesis of substances transported through ABC transporter.

### Overexpression of SerE and NCgl0581

**Fig. 5:**
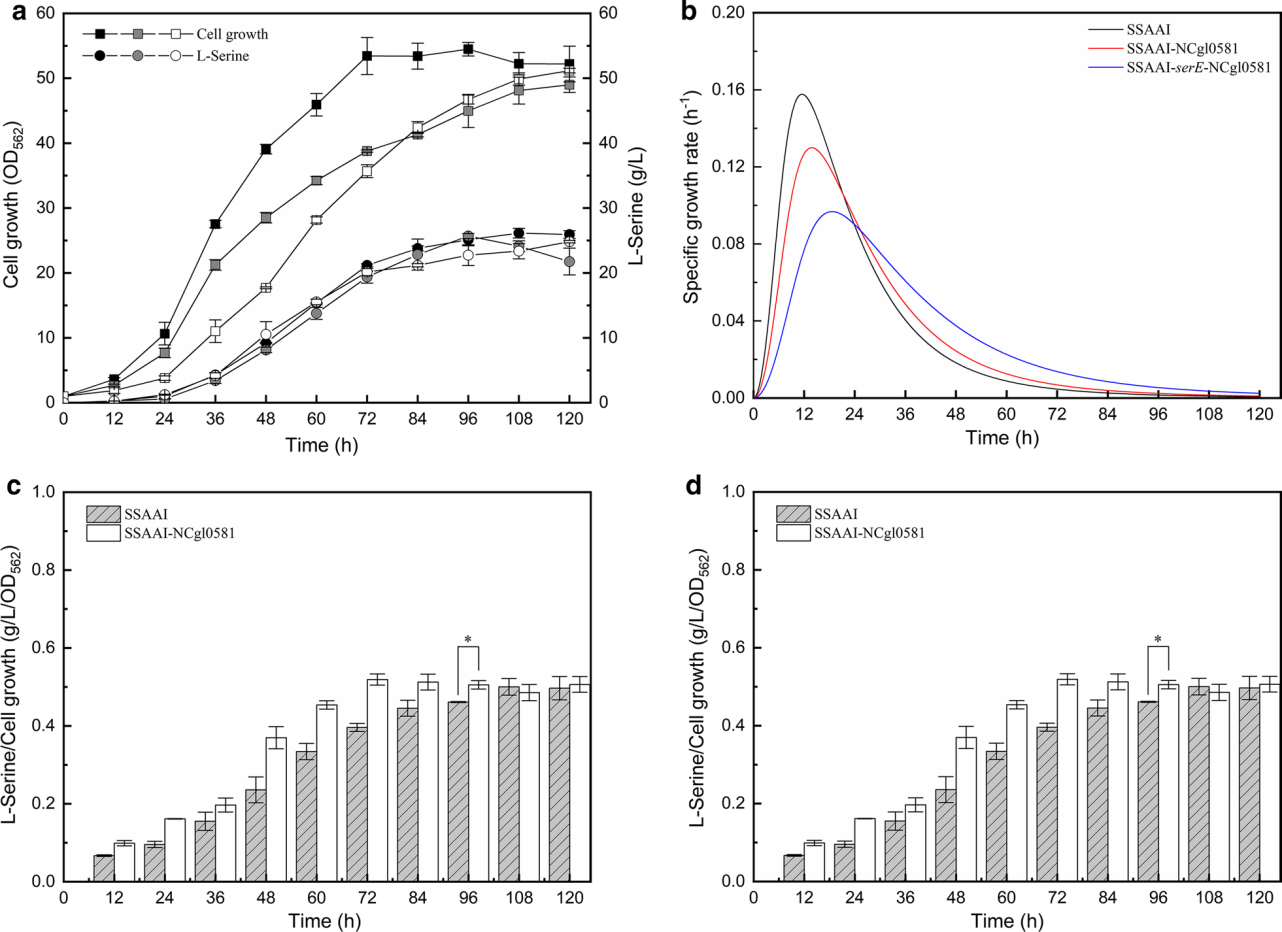
Effect of *serE* and NCgl0581 deletion or overexpression on SSAAI. **a** Cell growth (squares) and l-serine titer (circles) of SSAAI (black), NCgl0581 overexpression strain SSAAI-NCgl0581 (gray), and NCgl0581 and *serE* double overexpression strain SSAAI-NCgl0581-*serE* (white). **b** The growth rates of SSAAI-NCgl0581 (red), SSAAI-NCgl0581-*serE* (blue) and SSAAI (black). **c** Yp/x of SSAAI (gray bar with slash) and NCgl0581 overexpression strain SSAAI-NCgl0581 (white bar). **d** Yp/x of SSAAI (gray bar with slash) and NCgl0581 and *serE* double overexpression strain SSAAI-NCgl0581-*serE* (white bar)

As NCgl0581 could activate the expression of SerE in SSAAI, the overexpression of NCgl0581, *serE*, or their co-expression was studied, and strains SSAAI-NCgl0581 and SSAAI-NCgl0581-*serE* were constructed, respectively. As shown in Fig. 5a, b, a decrease in cell growth was observed in SSAAI-NCgl0581-*ser*E and SSAAI-NCgl0581 before 96 h of fermentation, and SSAAI-NCgl0581-*ser*E showed the lowest growth rate, the time courses for l-serine production were similar in all the strains. Furthermore, the yield of l-serine to biomass (Yp/x) increased in both SSAAI-NCgl0581-*serE* and SSAAI-NCgl0581 (Fig. 5c, d), suggesting that overexpression of a novel exporter SerE and its transcriptional regulator NCgl0581 was beneficial for l-serine efflux, but not for cell growth. Besides, SSAAI-NCgl0581-*serE* and SSAAI-NCgl0581 exhibited 9.67% (p < 0.05) and 19.17% (p < 0.01) higher Yp/x in 96 h, respectively, when compared with SSAAI. A similar decrease in cell growth was observed in SSAAI-*serE* (Fig. 3c); however, the l-serine titer was 28.67 g/L, which was 10.5% (p < 0.01) higher than that noted in SSAAI. This decrease in cell growth in the recombinant strain could be due to the transportation of the synthesized l-serine out of the cell, resulting in inadequate intracellular l-serine for cell growth. Therefore, our subsequent investigation involved replenishment of l-serine by overexpressing l-serine synthetic pathway key enzyme.

### High yield production of l-serine through SerE combined with synthetic pathway

To direct more flux to l-serine synthesis, l-serine exporter SerE and l-serine synthetic pathway key enzyme (containing a feedback insensitive *serA*Δ197, *serC,* and *serB* encoding the deregulated 3-phosphoglycerate dehydrogenase, phosphoserine phosphatase, and phosphoserine aminotransferase, respectively) were co-overexpressed in SSAAI to obtain SSAAI-*serE*-*serA*Δ197-*serC*-*serB*. The recombinant strain shared similar typical growth curves as the parent strain SSAAI, and achieved a final l-serine titer of 32.8 g/L, which was 22.1% (p < 0.001) higher than that noted in SSAAI.

**Fig. 6:**
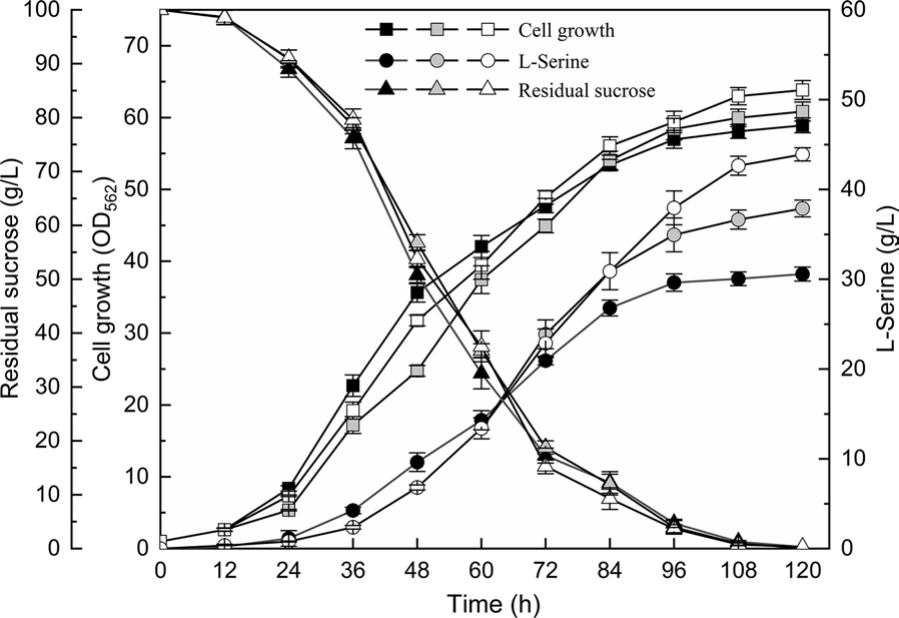
Fermentation process of strain A36 and strain A36-*serE*-*serA*Δ197-*serC*-*serB*. The cell growth (squares), l-serine titer (circles), and residual sucrose (triangles) of strain A36 (black), A36-*serA*Δ197-*serC*-*serB* (gray) and A36-*serE*-*serA*Δ197-*serC*-*serB* (white) are presented. Three parallel experiments were performed. Error bars indicate standard deviations of the results from three parallel experiments

Subsequently, to further improve l-serine titer, l-serine exporter *serE*, *serA*Δ197, *serC and serB* were overexpressed in strain A36, which was stemmed from SSAAI by using ARTP mutation [23]. As shown in Fig. 6, no significant changes can be observed for cell growth and sucrose consumption between strain A36-*serE*-*serA*Δ197-*serC*-*serB* and its control strain A36-*serA*Δ197-*serC*-*serB* as well as the parent strain A36. Interestingly, when the incubation time of batch cultivation exceeded 84 h, l-serine titer of A36-*serE*-*serA*Δ197-*serC*-*serB* were higher than A36-*serA*Δ197-*serC*-*serB.* After 120 h of cultivation, A36-*serE*-*serA*Δ197-*serC*-*serB* consumed all of the sucrose and achieved a final l-serine titer of 43.9 g/L, with a conversion rate of 0.44 g/g, which is 15.8% (p < 0.01) and 15.8% (p < 0.01) higher than the control strain A36-*serA*Δ197-*serC*-*serB* (37.9 g/L, 0.38 g/g), 43.5% (p < 0.001) and 41.9% (p < 0.001) higher than the parent strain A36 (30.6 g/L, 0.31 g/g), respectively. These results demonstrated that overexpression of l-serine exporter in combination with l-serine synthetic pathway could facilitate l-serine production in *C. glutamicum.*

## Discussion

Transport engineering is becoming an attractive strategy for strain improvement [11, 16, 17]. However, only a relatively limited number of exporters of amino acids have been identified in *C. glutamicum* (Additional file 1: Table S1) [8, 12, 14–17, 24–27]. In this study, SerE was identified as a novel l-serine exporter in *C. glutamicum*. Further analysis showed that SerE could also export l-threonine (Fig. 2c), but not l-cysteine, similar to ThrE, which could export both l-serine and l-threonine in *C. glutamicum* [15]. It was assumed that the presence of –OH in both l-serine and l-threonine might be the reason for these exporters to transport the two substrates. Based on homology search, SerE was found to be similar to a member of the DMT superfamily. Although DMT superfamily proteins are involved in the transport of a wide range of substrates, there are only a few reports available on their structures and mechanisms of substrate transport. Christian et al. performed structural and functional analyses of YddG, a DMT protein, and provided insight into the common transport mechanism shared among the DMT superfamily members [28]. It has been reported that analyses of the crystal structure data of exporters could help to elucidate the elusive transport mechanism [29], and in the future, we will further investigate the SerE structures and mechanisms of substrate transport.

To explore the interaction between the known l-serine exporter ThrE and the novel exporter SerE on l-serine export, ThrE and SerE single and double mutants were constructed. The results showed that *serE* and *thrE* double deletion mutant could still accumulate 10.34 g/L l-serine (Fig. 3b), suggesting that *C. glutamicum* might also possess other l-serine exporter systems. The evolution of multiple exporter systems for a single substrate is beneficial for the survival of bacteria in variable environment [7, 30]. It must be noted that overexpression of *serE* in SSAAI resulted in 10.5% (p < 0.01) increase in l-serine titer, but a decrease in cell growth. This could be due to the use of constitutive-type promoter to overexpress SerE, causing higher l-serine efflux. As sufficient l-serine content is important to maintain cell growth, a decrease in cell growth was noted as a stress response to *serE* overexpression. In future studies, better tuning of the *serE* expression must be achieved in SSAAI by testing different promoters and RBS. When *thrE* and *serE* were co-overexpressed in SSAAI, SSAAI-*serE*-*thrE* exhibited lower cell growth than SSAAI, but an l-serine titer similar to that of SSAAI-*serE* (Fig. 3c). A severe decline in cell growth was observed in all exporter overexpression strains, which may be caused by the accumulation of l-serine in the medium as well as additional burden on the cell overexpressing the exporters, similar to that reported by Mundhada et al. [31].

NCgl0581 was identified as the transcriptional regulator of the novel l-serine exporter SerE, and EMSA was performed to confirm the binding sites of NCgl0581 with the promoter of SerE. A previous study reported that the first member of the protein-gene pairs, ArgP-*argO* in *E. coli* and LysG-*lysE* in *C. glutamicum,* is a LysR-type transcriptional regulator, while the second member is its target gene encoding an amino acid exporter [22, 32, 33]. Similarly, NCgl0581-*serE* might also be a protein-gene pair sharing the same regulation mechanism. A serine biosensor based NCgl0581 was reported by Binder et al. [34], and accordingly, we constructed a biosensor for l-serine and found that NCgl0581 activated NCgl0580 (SerE) expression in the presence of l-serine, with expression of SerE enhancing with increasing l-serine titer [23]. However, NCgl0581 did not activate the expression of SerE in the presence of l-alanine and l-valine. To further confirm whether SerE could export l-alanine and l-valine, peptide feeding assays were employed using dipeptides (Ala–Ala, Val–Val) with SSAAI and SSAAI Δ*serE*. The results revealed that SerE could neither export l-alanine nor l-valine (data not shown). Moreover, transcriptome sequencing showed that NCgl0581 regulated 115 genes in *C. glutamicum*, suggesting that NCgl0581 was a novel global transcriptional regulator in *C. glutamicum*. Transcriptional regulators and their roles in expression control of target genes are important for metabolic engineering of *C. glutamicum* for industrial applications [35], and the present study provided a new member of transcriptional regulator family.

Overexpression of SerE alone in SSAAI resulted in 10.8% increase in l-serine titer and a simultaneous decrease in cell growth, implying that the synthesized l-serine was transported out of the cell, and that the intracellular l-serine was not adequate for cell growth. When l-serine was replenished by overexpressing l-serine synthetic pathway key enzyme, the cell growth was restored and l-serine titer increased to 43.9 g/L, with an l-serine yield of 0.44 g/g sucrose, which are the highest yield reported so far for *C. glutamicum*. These results indicated that *serA*Δ197, *serC*, and *serB* overexpression ensured sufficient l-serine supply preventing cell growth inhibition. In previous studies by Mundhada et al. 37 g/L l-serine was produced with a yield of 0.24 g/g glucose in *E. coli* [2], and 11.7 g/L l-serine titer was achieved with the highest yield of 0.43 g/g glucose [31]. Interestingly, in the present study, we found that the l-serine titer significantly increased with *serB* overexpression in A36, producing an l-serine titer of 37.9 g/L, which was 24% higher than that in A36. It must be noted that *serB* encodes phosphoserine phosphatase (PSP, EC 3.1.3.3), which catalyzes the last step of l-serine biosynthesis. However, l-serine titer did not significantly change when *serA*Δ197 and *serC* were respectively overexpressed in A36 (with l-serine titer of 31.1 and 32.78 g/L, respectively). In a recent study, 50 g/L l-serine was produced with glucose as the carbon source in *E. coli*, which is the highest l-serine production reported so far; however, the yield was 0.36 g/g glucose [36], which is lower than that obtained in the present study (0.44 g/g sucrose). Therefore, fine controlling of the three enzymes of l-serine biosynthesis pathway could possibly further enhance l-serine production.

## Conclusion

In the present study, a novel exporter SerE and its positive regulator NCgl0581 were identified in *C. glutamicum.* SerE exhibited the ability to export l-threonine and NCgl0581 acting as a novel global transcriptional regulator in *C. glutamicum*, and by overexpressing this novel exporter along with l-serine synthetic pathway enzyme, significant increase in l-serine yield could be achieved. These results enrich our understanding of amino acid transport and can provide additional targets for exporter engineering in *C. glutamicum*.

## Materials and methods

### Strains, plasmids, and growth conditions

The strains and plasmids used in this study are listed in Table 3. *E. coli* JM109 was used as the cloning host, and was grown in lysogeny broth (LB) medium (containing 5.0 g/L yeast extract, 10.0 g/L tryptone, and 10.0 g/L NaCl) at 37 °C and 220 rpm. The engineered SSAAI (CGMCC No.15170) was selected as the original strain, which constructed in our laboratory by knocking out 591 bp of the C-terminal domain of *serA*, deleting *sdaA*, *avtA*, and *alaT*, as well as attenuating *ilvBN* in the genome of *C. glutamicum* SYPS-062-33a (CGMCC No. 8667). Strain A36 derived from SSAAI by ARTP mutation, with higher l-serine titer and yield than SSAAI. The seed and fermentation media for *C. glutamicum* were prepared as described previously [5]. The *C. glutamicum* strains were pre-incubated in the seed medium overnight to an optical density (OD_562_) of about 25, and then inoculated at an initial concentration of OD_562_ = 1 into a 250 mL flask containing 25 mL of the fermentation medium at 30 °C and 120 rpm. The antibiotic kanamycin (50 mg/L) was added when necessary. Samples were withdrawn periodically for the measurement of residual sugar, amino acids, and OD_562_ as described previously [5].

**Table 3:** Strains and plasmids used in this study

Strain/plasmid	Description	Sources or reference
*E. coli*		
JM109	*recA*1, *endA*1, *gyrA*96, *thi*-1, *hsdR*17, *supE*44, *relA*1	Laboratory strain
*C. glutamicum*		
SSAAI	*C. glutamicum* SYPS-33a with deletion of the 591 bp in the C-terminus of *serA*, deletion of *sdaA*,*alaT*,*avta* and attenuation of *ilvBN*	[5]
A36	SSAAI mutant strain	[23]
SSAAI-*thrE*	SSAAI harboring plasmid pDXW-10-*thrE*	This study
SSAAIΔ*thrE*	SSAAI with deletion of *thrE*	This study
SSAAIΔNCgl2050	SSAAI with deletion of NCgl2050	This study
SSAAIΔNCgl2065	SSAAI with deletion of NCgl2065	This study
SSAAIΔNCgl0580	SSAAI with deletion of NCgl0580	This study
SSAAI-10	SSAAI harboring plasmid pDXW-10	This study
SSAAI-*egfp*	SSAAI harboring plasmid pDXW-10-*egfp*	This study
SSAAI-*serE*-*egfp*	SSAAI harboring plasmid pDXW-10-*serE*-*egfp*	This study
SSAAI-NCgl0581	SSAAI harboring plasmid pDXW-10- NCgl0581	This study
SSAAI-NCgl0581-*serE*	SSAAI harboring plasmid pDXW-10- NCgl0581-*serE*	This study
SSAAIΔNCgl0581	SSAAI with deletion of NCgl0581	This study
SSAAIΔNCgl0581-1	SSAAIΔNCgl0581 harboring pDXW-11-1	This study
SSAAIΔNCgl0581-0	SSAAIΔNCgl0581 harboring pDXW-11-0	This study
SSAAIΔNCgl0580- NCgl0580	SSAAIΔ*serE* harboring plasmid pDXW-10-*serE* (NCgl0580)	This study
SSAAI-*serE*	SSAAI harboring plasmid pDXW-10-*serE* (NCgl0580)	This study
ATCC13032	Wild type	Laboratory strain
ATCC13032Δ*serE*	ATCC13032 with deletion of *serE* (NCgl0580)	This study
pK18mob*sacB*	Integration vector, *oriV*, *oriT*, *mob*, *sacB*, Km^r^	[37]
pK18mob*sacB*Δ*thrE*	pK18mob*sacB* carrying the up- and downstream homologous fragments of *thrE* gene for *thrE* deletion	This study
pK18mob*sacB*ΔNCgl2050	pK18mob*sacB* carrying the up- and downstream homologous fragments of NCgl2050 gene for NCgl2050 deletion	This study
pK18mob*sacB*ΔNCgl2065	pK18mob*sacB* carrying the up- and downstream homologous fragments of NCgl2065 gene for NCgl2065 deletion	This study
pK18mob*sacB*ΔNCgl0580	pK18mob*sacB* carrying the up- and downstream homologous fragments of NCgl0580 gene for NCgl0580 deletion	This study
pK18mob*sacB*ΔNCgl0581	pK18mob*sacB* carrying the up- and downstream homologous fragments of NCgl0581 gene for NCgl0581 deletion	This study
pDXW-10	*E. coli*-*C. glutamicum* shuttle vector, *tacM* promoter, Km^r^	[38]
pDXW-10-*thrE*	pDXW-10 carrying the gene of *thrE*	This study
pDXW-10-*serE*	pDXW-10 carrying the gene of *serE*	This study
pDXW-10-*egfp*	pDXW-10 carrying the gene of *egfp*	This study
pDXW-10-*egfp*-*serE*	pDXW-10 carrying the gene of *egfp and serE* for the expression of fusion protein EGFP-SerE	This study
pDXW-10- NCgl0581	pDXW-10 carrying the gene of NCgl0581	This study
pDXW-10- NCgl0581-*serE*	pDXW-10 carrying the gene of NCgl0581 and *serE*	This study
pDXW-11	*E. coli*-*C. glutamicum* shuttle vector, probe plasmid, Km^r^	[39]
pDXW-11-1	pDXW-11 carrying the fragments of NCgl0581, the intergenic region between NCgl0581 and NCgl0580, and *egfp*	This study
pDXW-11-0	pDXW-11 carrying the fragments of the intergenic region between NCgl0581 and NCgl0580, and *egfp*	This study

*Km*^*r*^ kanamycin resistance

### Construction of plasmids and strains

The primers used in this study for gene expression/deletion are listed in Additional file 1: Table S2. Gene deletion was performed using the nonreplicable deletion vector pK18mob*sacB*, as reported previously [37]. For example, to achieve *thrE* deletion, the homologous-arm fragments for *thrE* deletion were amplified from SSAAI chromosome using the primer pairs *thrE*1/2 for the upstream fragment and *thrE*3/4 for the downstream fragment. Then, with the two fragments as templates, a crossover PCR was performed using the primer pair *thrE*1/4. The truncated product of *thrE* was digested with *Xba*I and *Hind*III and ligated to the vector pK18mob*sacB* that was similarly treated. The recombinant plasmid pK18mob*sacB*Δ*thrE* was transformed into SSAAI competent cells by electroporation, and chromosomal deletion was performed by selecting cells that were kanamycin resistant and sucrose nonresistant, and verified by PCR.

The pDXW-10 and pDXW-11 plasmids were used to overexpress genes in *C. glutamicum* [38, 39]. The recombinant plasmids were constructed as follows: the genes *thrE* and *serE* were amplified, digested, and ligated to the pDXW-10 plasmid that was digested with *Hind*III/*Bgl*II. The plasmid harboring the fusion protein, EGFP-SerE (enhanced green fluorescent protein), was constructed by using the method reported in a previous study [19]. To confirm the role of NCgl0581 on NCgl0580 expression, the fragment consisting of intergenic region of NCgl0581 and NCgl0580 and EGFP with or without NCgl0581 was ligated to the plasmid pDXW-11 by Clon Express MultiS One Step Cloning Kit (Vazyme, Nanjing, China). The strains were constructed by electroporation with the corresponding plasmids.

The genes, *serA*Δ197, *serC*, and *serB*, were PCR amplified from SSAAI using primers shown in Additional file 1: Table S2. To construct plasmid pDXW-10-*serA*Δ197, the resultant fragment of *serA*Δ197 was digested with *EcoR*I and *Not*I and cloned into pDXW-10. To construct plasmid pDXW-10-*serA*Δ197-*serC*-*serB*, the PCR fragments of *serB* and *serC* were digested with appropriate restriction enzymes and successively cloned into the corresponding plasmids to form plasmid pDXW-10-*serA*Δ197-*serC*-*serB.* The resulting plasmid (pDXW-10-*serA*Δ197-*serC*-*serB*) was then subjected to double digestion by *Nde*I and *Pac*I for cloning of NCgl0580 to obtain pDXW-10-*serE*-*serA*Δ197-*serC*-*serB*.

### Confocal microscopic observation

The strains SSAAI-10 (SSAAI-harboring plasmid pDXW-10), SSAAI-*egfp*, and SSAAI-*serE*-*egfp* were grown in the seed medium and harvested during the exponential phase. The cells were washed twice and maintained in PBS (pH 7.4), mounted on a microscope slide, and observed under a Leica laser scanning confocal microscope (Leica, TCS SP8; Leica, Wetzlar, Germany) equipped with a HC PL Apo 63x/1.40 Oil CS2 oil-Immersion objective, with excitation filter at 488 nm and emission filter at 510–550 nm. The digital images were acquired and analyzed with Lecia Application Suite X 2.0.

### Membrane and cytoplasmic protein extraction and fluorescence measurements

The strains SSAAI-10, SSAAI-*egfp*, and SSAAI-*serE*-*egfp* were used for extracting membrane and cytoplasmic proteins to determine SerE localization. The extraction was performed using Membrane and a Cytoplasmic Protein Extraction Kit according to the manufacturer’s protocol (Beyotime, Nanjing, China). The cells were washed twice with PBS (pH 7.4) and disrupted by ultrasonication on ice (pulse, 4 s; interval, 6 s; total duration, 30 min) (Sonics Vibra-Cell™, Sonics, Newtown, CT, USA). The supernatant containing cytoplasmic proteins was collected by centrifugation (700×*g*, 4 °C for 10 min), and the precipitate was used for extracting membrane proteins. The protein concentration was determined with a Modified BCA Protein Assay Kit (Sangon, China). After extraction, the fluorescence intensity (excitation at 488 nm, emission at 517 nm) of the membrane and cytoplasmic proteins was determined using fluorescence spectrophotometer (Synergy H4; BioTek, Winooski, VT, USA).

### Amino acid export assay

For ascertaining the function of *serE*, a dipeptide Ser–Ser addition assay was performed [15]. In brief, the pre-incubated cells (in seed medium) were washed once with CGXII minimal medium [40], inoculated into CGXII minimal medium with 2 mM Ser–Ser (other dipeptide), and incubated for 2 h at 30 °C. Then, the cells were harvested, washed once with cold CGXII minimal medium, and resuspended in CGXII minimal medium. Amino acid excretion was initiated by adding 2 mM Ser–Ser (other dipeptide). HPLC was used to determine the concentrations of amino acids [19].

### Analytical procedures

Cell density (OD_562_) was measured using an AOE UV-1200S UV/vis spectrophotometer (AOE Instruments Co. Inc., Shanghai, China). Sugar concentration was determined using SBA-40E glucose analyzer (Biology Institute of Shandong Academy of Sciences, China). For measurement of extracellular l-serine concentration in shake-flask fermentation, 500 μL of the culture were centrifuged at 700×*g* for 5 min, and the supernatant was used for detection after appropriate dilution. To ascertain intracellular l-serine concentration, 300 μL of the culture were centrifuged at 700×*g* and 4 °C for 10 min, and 300 μL of water were added to the cells. The cells were disrupted by FastPrep-24 5G instrument (5 m/s, 120 s, MP Biomedicals, Shanghai, China). The cytoplasmic volume was assumed to be 2 μL/mg dry cell weight [27]. The titers of intracellular and extracellular l-serine and other amino acids were analyzed by HPLC using phenyl isothiocyanate as a precolumn derivatization agent, according to our previously study [8].

### EMSA

To identify the binding site of NCgl0581 in the NCgl0580 promoter region, EMSA was conducted by using Non-Radioactive EMSA Kits with Biotin-Probes User’s Manual VER. 5.11 (Viagene Biotech Inc, Changzhou, China), according to the manufacturer’s instruction. The consensus oligonucleotides were BIO-JNZXM-TP (5′-AAACAGCCAA CTATAGTTAAGTAATA-3′) and BIO-JNZXM-BM (5′-TATTACTTAACTATAGTTGGCTGTTT-3′). The positive control was the nuclear extracts with activated specific TF, and the negative control was the nuclear extracts without activated TF.

## Supplementary information

**Additional file 1.** Additional tables and figures.

## Data Availability

The datasets used and analyzed in this study are available from the corresponding author on request.

## Abbreviations

*C. glutamicum*: *C. glutamicum*
SSAAI: *C. glutamicum* ΔSSAAI
EGFP: Enhanced green fluorescent protein
EMSA: Electrophoretic mobility shift assays
DMT: Drug/metabolite transporter superfamily
Thr-Thr: L-Threonine dipeptides
Cys-Cys: L-Cysteine dipeptides
Ala-Ala: L-Alanine dipeptides
Val-Val: L-Valine dipeptides
LTTRs: LysR-type transcriptional regulators family
Yp/x: Yield of l-serine to biomass
PSP: Phosphoserine phosphatase
LB: Lysogeny broth
ARTP: Atmospheric and room temperature plasma
