# Supplementary material for: High-yield production of l-serine through a novel identified exporter combined with synthetic pathway in Corynebacterium glutamicum
Source: Microb Cell Fact. 2020 May 29;19:115. doi: 10.1186/s12934-020-01374-5 (PMC7260847; doi:10.1186/s12934-020-01374-5)
Supplement: Supplementary file 1 — Additional file 1. Additional tables and figures. [file 12934_2020_1374_MOESM1_ESM.docx]

Additional Tables

**TABLE S1 The studies of amino acid exporter in *C. glutamicum***

| **Exproter** | **Amino acid substrate** | **Family** | **Comments** | **Reference** |
| --- | --- | --- | --- | --- |
| AroP | L-Phenylalanine,  L-Tryptophan, L-tyrosine, L-Histidine | APC | Histidine uptake by AroP is competitively inhibited by the aromatic amino acids. | [1] |
| BrnFE | L-Valine, L-Leucine, L-Isoleucine, L-Methionine | LIV-E | Transcriptionally controlled by the activator Lrp which is dependent on L-valine, L-leucine, L-isoleucine, and L-methionine | [2, 3] |
| CgmA | L-Arginine | MFS | Transcriptionally controlled by the repressor CgmR which is dependent on diamines | [4] |
| LysE | L-Lysine, L-Arginine,  L-Citrulline | LysE | Transcriptionally controlled by the activator LysG which is dependent on the basic amino acids L-lysine, L-arginine, L-histidine, and L-citrulline | [5] |
| MscCG2 | L-Glutamate | MscS | MscCG2-mediated L-glutamate excretion was activated by biotin limitation or penicillin treatment. | [6] |
| PheP | L-Phenylalanine | APC | The uptake of L-The was not inhibited by nine amino acids but by L-Tyr. | [7] |
| ThrE | L-Threonine, L-Serine | ThrE | The carrier encoded by ThrE serves to export small molecules such as L-threonine and L-serine | [8, 9] |
| YggB | L-Glutamate | MscS | Osmo regulated channel | [10] |

**TABLE S2 Primes used in this study.**

| **Primer** | **Sequences (5'-3')** | **Purpose** |
| --- | --- | --- |
| *thrE-*1 | GCTCTAGACATCAATCTGGTCAACGAA (*Xba*I) | *thrE*-1 to *thrE*-4 for *thrE* deletion |
| *thrE-*2 | **GACATGGAGATGAGCTAAG**AATGCGGCCACGAAGGGTC |  |
| *thrE-*3 | **CTTAGCTCATCTCCATGTC**TCAACCCATACCGTGCATT |  |
| *thrE-*4 | CCCAAGCTTATCATCCATATAAGATCCG (*Hind*Ⅲ) |  |
| 2050-1 | CGGAATTCCTAGCTGGCCTTCGTACGAT (*EcoR*I) | 2050-1 to 2050-4 for NCgl2050 gene deletion |
| 2050-2 | **TGGACGAAGCCTAGACAAC**GCAATTACGCCGTAGATCAT |  |
| 2050-3 | **GTTGTCTAGGCTTCGTCCA**GCGGTGGCGATCTATCTCACCG |  |
| 2050-4 | GCTCTAGAGTGACATGCTCGGCCAAGAC (*Xba*I) |  |
| 2065-1 | CGGAATTCTGGTGAGGAAGCTGTTGCAT (*EcoR*I) | 2065-1 to 2065-4 for NCgl2065 gene deletion |
| 2065-2 | **AGACAGACATGTGGAGACC**CACGCCGTTAACCACCATCA |  |
| 2065-3 | **GGTCTCCACATGTCTGTCT**TCCTAGCGGTTTCCCACAC |  |
| 2065-4 | CCCAAGCTTCCGAGGAGGGTAAGCCAGT (*Hind*Ⅲ) |  |
| 0580-1 | TCCCCCGGGTTCGAGCGCTGCGGTGACT (*Sma*I) | 0580-1 to 0580-4 for NCgl0580 gene deletion |
| 0580-2 | **GTAGACATGACGGCGACTT**TGCAGGGATAGGGCGGAAC |  |
| 0580-3 | **AAGTCGCCGTCATGTCTAC**GTGGGCCGCGATCATCCTT |  |
| 0580-4 | GCTCTAGAATGTTCCTGTCATCGCTGG (*Xba*I) |  |
| 0581-1 | CGGAATTCGCCGCTGAGGGAATCGATAC (*EcoR*I) | 0581-1 to 0581-4 for NCgl0581 gene deletion |
| 0581-2 | **CTGGAGAGCCTAGATACGC**CGGTTGAGATTGAGCACGAC |  |
| 0581-3 | **GCGTATCTAGGCTCTCCAG**GGAGGCTCGTCTAGTAGAGT |  |
| 0581-4 | GCTCTAGACTTCGGAGGACGCGGTGACT (*Xba*I) |  |
| *thrE*-F | GAAGATCTAGAAGGAGATATACCATGTTGAGTTTTGCGACCCT (*Bgl*Ⅱ) | *thrE*-F and *thrE*-R for *thrE* amplication |
| *thrE*-R | CCCAAGCTTTTACCTTTTATTACCGAATC (*Hind*Ⅲ) |  |
| *serE*-F | GAAGATCTAGAAGGAGATATACCATGAATAAACAGTCCGCTGC (*Bgl*Ⅱ) | *serE*-F and *serE*-R for *serE* amplication |
| *serE*-R | CCCAAGCTTTTAACTAGGTGTGTGTACTC (*Hind*Ⅲ) |  |
| *egfp*-F | GAAGATCTAGAAGGAGATATACCATGGTGAGCAAGGGCGAG (*Bgl*Ⅱ) | *egfp*-F and *egfp*-R for *egfp* amplication |
| *egfp*-R | CCCAAGCTTTTACTTGTACAGCTCGTC (*Hind*Ⅲ) |  |
| NCgl0581-F | cgcctcgagggatccagatctTCACTCTACTAGACGAGCCTCCAA | NCgl0581-F and NCgl0581-R for NCgl0581 gene amplication |
| NCgl0581-R | catccgccaaaacagaagcttGTGCTCAATCTCAACCGCTTACA |  |
| NCgl0581-serE-F | cgcctcgagggatccagatctTCACTCTACTAGACGAGCCTCCAA | NCgl0581-serE-F and NCgl0581-serE-R for NCgl0581-serE amplication |
| NCgl0581-serE-R | catccgccaaaacagaagcttTTAACTAGGTGTGTGTACTCGCCTC |  |
| *serE-egfp*-1 | GAAGATCTAGAAGGAGATATACCATGAATAAACAGTCCGCTGC (*Bgl*Ⅱ) | *serE-egfp*-1 to *serE-egfp*-4 for expression of fusion protein SerE-EGFP |
| *serE-egfp*-2 | **GCTACCGCTACCGCTACCGCTACC**GGTGTGTGTACTCGCCTC |  |
| *serE-egfp*-3 | **ACACCGGTAGCGGTAGCGGTAGCGGTAGC**ATGGTGAGCAAGGGCGAG |  |
| *serE-egfp*-4 | CCCAAGCTTTTACTTGTACAGCTCGTC (*Hind*Ⅲ) |  |
| 11-1-1 | gtggtggtggtggtgctcgagTTACTTGTACAGCTCGTCCATGCC | 11-1-1 to 11-1-4 for the construction of pDXW-11-1 |
| 11-1-2 | attggaacgcATGGTGAGCAAGGGCGAGG |  |
| 11-1-3 | tgctcaccatGCGTTCCAATGGCAGCAC |  |
| 11-1-4 | tctggatccgaattcgagctcTCACTCTACTAGACGAGCCTCCAA |  |
| 11-0-1 | gtggtggtggtggtgctcgagTTACTTGTACAGCTCGTCCATGCC | 11-0-1 to 11-0-4 for the construction of pDXW-11-0 |
| 11-0-2 | attggaacgcATGGTGAGCAAGGGCGAGG |  |
| 11-0-3 | tgctcaccatGCGTTCCAATGGCAGCAC |  |
| 11-0-4 | tctggatccgaattcgagctcGACACCTCCAAAATTGTTCAGTATTAC |  |
| *serA*-F | CGGAATTCGAAAGGAATATACCGTGAGCCAGAATGGCCGTC (*Eco*R I) | *serA*-F and *serA* -R for *serA* amplication |
| *serA*-R | GAAGATCTTTAAGCCAGATCCATCCAC (*Bgl* II) |  |
| *serC*-F | CGGAATTCGAAAGGAATATACCATGACCGACTTCCCCACCCTG (*Eco*R I) | *serC*-F and *serC*-R for *serC* amplication |
| *serC*-R | GAAGATCTTTACTTCTTTGCAAAACCGCC (*Bgl* II) |  |
| *serB*-F | CGGAATTCGAAAGGAATATACCGTGACTGAACTCATCCAGAA (*Eco*R I) | *serB*-F and *serB*-R for *serB* amplication |
| *serB*-R | CCCAAGCTTTTAGGCATTGGTCAATGGAAC (*Hind* III) |  |
| *serC*-3F | GCTCTAGACTGAAATGAGCTGTTGACAA (*Xba* I) | *serC*-3F to *serB*-3R for *serA,* *serB* and *serC* amplication |
| *serC*-3R | CCGCTTAAGTTACTTCTTTGCAAAACCGC (*Afl* II) |  |
| *serB*-3F | GAAGATCTCTGAAATGAGCTGTTGACAA (*Bgl* II) |  |
| *serB*-3R | GCTCTAGATTAGGCATTGGTCAATGGAA (*Xba* I) |  |

Restriction sites are underlined; overlapping sequences are bold letters; the sequences of homologous recombination are shown with lowercase letter.

Additional Figures


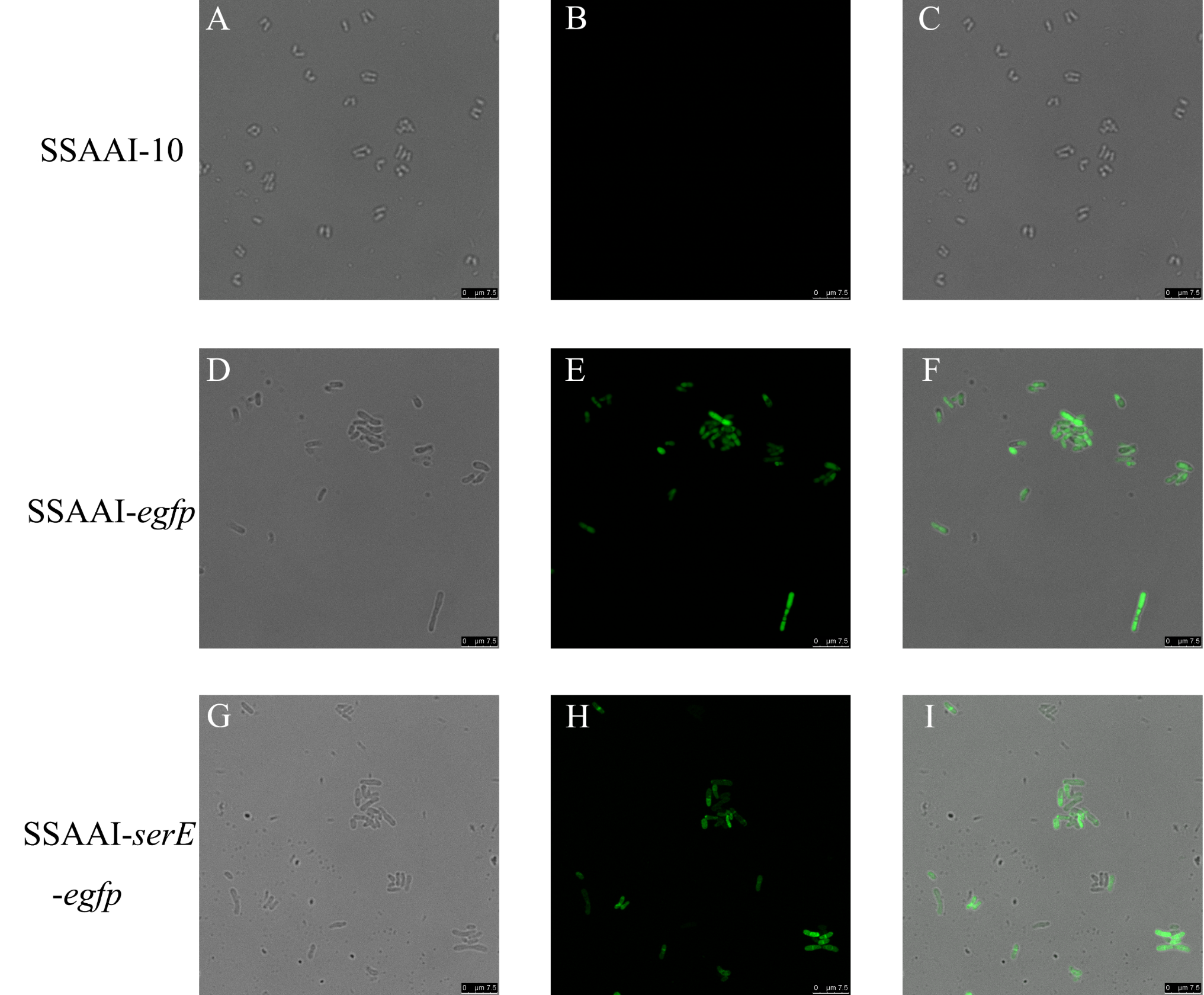


**Fig S1 Confocal microscopic evaluation of protein expression.**

SSAAI-10 (SSAAI harboring plasmid pDXW-10 only, A-C), SSAAI-*egfp* (SSAAI expressing EGFP protein with pDXW-10, D-F), and SSAAI-*serE*-*egfp* (SSAAI expressing SerE-EGFP fusion protein with pDXW-10, G-I) observed under visible light (A, D, and G) and excitation filter at 488 nm (B, E, and H). Images C, F, and I were stacked under visible light and excitation filter at 488 nm. All of the images were generated under the same intensities of excitation.


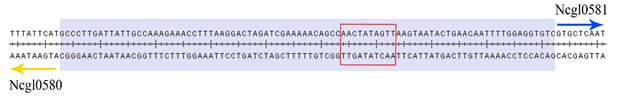


**Fig S2 The location of NCgl0581 gene.**

The gene NCgl0581 divergently transcribed from *serE* and located upstream of *serE*.

**REFERENCES**

1. Shang X, Zhang Y, Zhang G, Chai X, Deng A, Liang Y, Wen T. Characterization and molecular mechanism of AroP as an aromatic amino acid and histidine transporter in *Corynebacterium glutamicum*. J Bacteriol. 2013; 195:5334-5342.

2. Schwede T. Protein modeling: what happened to the "Protein Structure Gap"? Structure. 2013; 21:1531-1540.

3. Kennerknecht N, Sahm H, Yen MR, Patek M, Saier MH, Eggeling L. Export of L-isoleucine from *Corynebacterium glutamicum*: A two-gene-encoded member of a new translocator family. J Bacteriol. 2002; 184:3947-3956.

4. Lubitz D, Jorge JMP, Perez-Garcia F, Taniguchi H, Wendisch VF. Roles of export genes *cgmA* and *lysE* for the production of L-arginine and L-citrulline by *Corynebacterium glutamicum*. Appl Microbiol Biotechnol. 2016; 100:8465-8474.

5. Vrljic M, Sahm H, Eggeling L: A new type of transporter with a new type of cellular function: L-lysine export from *Corynebacterium glutamicum*. Mol Microbiol. 1996; 22:815-826.

6. Wang Y, Cao G, Xu D, Fan L, Wu X, Ni X, Zhao S, Zheng P, Sun J, Ma Y. A novel L-glutamate exporter of *C. glutamicum*. Appl Environ Microb. 2018; 84(6): 15

7. Zhao Z, Ding J, Li T, Zhou N, Liu S. The NCgl1108 (PheP (Cg)) gene encodes a new L-Phe transporter in *C. glutamicum*. Appl Microbiol Biotechnol. 2011; 90:2005-2013.

8. Simic P, Sahm H, Eggeling L. L-threonine export: Use of peptides to identify a new translocator from *Corynebacterium glutamicum*. J Bacteriol. 2001; 183:5317-5324.

9. Eggeling L, Sahm H. New ubiquitous translocators: amino acid export by *Corynebacterium glutamicum* and *Escherichia coli*. Arch Microbiol. 2003; 180:155-160.

10. Nakamura J, Hirano S, Ito H, Wachi M. Mutations of the *Corynebactetium glutamicum* NCgl1221 gene, encoding a mechanosensitive channel hornolog, induce L-glutarnic acid productionv. Appl Environ Microb. 2007; 73:4491-4498.
